# Can patient-reported outcome measures be used to predict consultation needs in patients with psoriasis?: A survey study

**DOI:** 10.1186/s41687-022-00490-7

**Published:** 2022-07-23

**Authors:** Anna Sophie Belling Krontoft, Johanna Walsøe Jensen, Mette Charlotte Pedersen, Maria Pors, Diljit Kaur-Knudsen, Claus Zachariae, Lone Skov

**Affiliations:** Department of Dermatology and Allergy, Herlev and Gentofte Hospital, University of Copenhagen, Hellerup, Denmark

**Keywords:** PROMs, Psoriasis, Patient-reported outcomes, Visitation, Consultation needs

## Abstract

**Background:**

Patient-reported outcome measures (PROMs) are emerging tools used to capture a patient’s daily health status and enhance communication between patients and healthcare professionals. This study examined whether PROMs can be used to predict consultation needs in an outpatient clinic setting including patients diagnosed with psoriasis.

**Method:**

We evaluated a nationally developed set of PROMs for psoriasis patients, which included a standard set of questionnaires that capture patients’ perceptions of their experience and quality of life. Patients (n = 187) answered the psoriasis PROMs prior to an in-person consultation. Their responses were evaluated alongside patient, doctor, and nurse opinions on whether the subsequent consultation was necessary. Additionally, comments about the consultations from the patient, doctor, and nurse were collected and provided insights as to why certain consultations were deemed necessary.

**Results:**

Comparing the patient, doctor, and nurse responses addressing a need for consultation compared to the coded psoriasis PROMs results (red or green/yellow outcome), 23% of the patients with a green/yellow outcome were in need of a doctor’s consultation. Upon considering a subset of psoriasis PROMs questionnaires that reflect subjective responses (e.g., DLQI, PEST, MDI-2, and side effects), the proportion of patients that yielded a green/yellow outcome and were identified to require a doctor consultation increased to approximately 45%.

**Conclusions:**

The preliminary results show that the psoriasis PROMs were supportive in the consultation but alone cannot sufficiently guide healthcare professionals to determine whether in-person consultations are required.

**Supplementary Information:**

The online version contains supplementary material available at 10.1186/s41687-022-00490-7.

## Background

Psoriasis is a chronic inflammatory skin disease that impacts 3–4% of adults worldwide. In Scandinavia, the estimated prevalence of psoriasis is higher than broad global estimates, affecting between 2.2 and 11.4% of adults [1]. Psoriasis impacts both the physical and psychological well-being of the patient. The physical symptoms associated with psoriasis include itching, pain, scaling plaques, and potential comorbidities such as coronary heart diseases and psoriasis arthritis. Patients with psoriasis also experience detrimental psychological and socioemotional effects that can reduce health related quality of life including altered body image and an increased risk of anxiety and depression [2, 3]. In a study from 2020 about stigma in psoriasis it is reported that two thirds of patients with psoriasis anticipated others to regard them as “contagious” and this often-prompted social avoidance with great consequences for the patients psychological well-being [4].

Psoriasis can present itself in different ways; plaque psoriasis, guttate psoriasis, pustular psoriasis etc., however the most common form is plaque psoriasis which affects approximately 80–90% of patients with psoriasis [3, 5]. The patients treated in the department most often suffer from plaque psoriasis in a moderate to severe degree.

Psoriasis activity fluctuates across a patient’s lifetime, changing the need for physician-based consultations over time. As such, patients may require more frequent consultations during periods of greater symptom severity and less frequent, time-consuming consultations during periods of remission [6]. Overall, a failure to deliver the right care at the right time accounts for roughly 40% of healthcare spending and represents one of the great challenges facing healthcare systems [2]. This demonstrates the importance of value-based healthcare that directs resources to improving outcomes and meeting patients’ needs.

In recent years, patient-reported outcome measures (PROMs) have allowed healthcare professionals (HCPs) to track patient experience between appointments to evaluate various aspects of their daily health and well-being [7]. The purpose of PROMs is to capture data that detail symptoms, functional status, and quality of life directly from the patient at regular intervals. This information can be used by HCPs to improve disease management, tailor individualized treatment plans, enhance patient and HCP communication, and increase overall patient satisfaction [8, 9]. By implementing PROMs within a value-based healthcare approach, this tool can transform patient-centred care [10].

In the present study, we examined a nationally developed set of PROMs designed for patients with psoriasis and the use for visitation support. Due to continual changes in severity and treatment needs, we hypothesize that patients with psoriasis and their HCPs will benefit from introducing PROMs into their treatment plans. The aim of this study was to use a set of PROMs that best supports psoriasis patients, and to evaluate whether this tool can be successfully used in an outpatient clinic setting to predict consultation needs.

## Methods

### Study design

A set of patient-reported outcome measures for patients with psoriasis (PSO PROMs) was developed in Denmark between 2019 and 2021 by a national clinical coordination group led by the Danish Health Data Authority. The PSO PROMs were tested in two departments of dermatology (one being the Department of Dermatology and Allergy, Herlev-Gentofte Hospital, Denmark) and several private practices [11], between December 2020 and June 2021. Simultaneously with the test of PSO PROM we, at the Department of Dermatology and Allergy, Herlev-Gentofte hospital, Denmark, carried out the PSO PROMs Visitation Study that investigated whether the PSO PROMs could be used to identify patients that require an in-person consultation. Here only the findings from the PSO PROMs Visitation study are presented.

### Study sample

Our study sample included patients over 18 years of age with a confirmed psoriasis diagnosis. The patients included had been treated at the study site for between a few months and up to several years. Our only exclusion criteria were newly referred patients being seen for the first time in the clinic and patients that did not speak and understand danish. Patients received a link to the PSO PROM questionnaire with their consultation booking confirmation. A PSO PROM link was sent to the patients and directed them to a website (MinSP) that is a part of the patient’s record (EPIC). The patients answered the PROMs online and when finished the answers would automatically be transferred to their patient record for the HCP to see. In Denmark survey studies does not acquire approval from the ethics committee and therefore it was not necessary to collect consents prior to the collection of PROM data. That said, all patients were told that participation was voluntary and if they chose not to participate it would have no consequences for their future treatment in the clinic. Patients who completed the PSO PROMs prior to their in-person visit were invited to participate in the PSO PROMs Visitation Study.

### The PSO PROMs

The PSO PROMs consisted of six questionnaires: (1) Dermatology Life Quality Index (DLQI) [12]; (2) Psoriasis Sign Score (PSSD sign) [13]; (3) Psoriasis Symptom Score (PSSD symptom) [13]; (4) Body Surface Area (BSA); (5) Major Depression Inventory (MDI-2) [14] and (6) Psoriasis Epidemiology Screening Tool (PEST) [15]. In addition to these standard assessments, the national clinical coordination group included three standard questions addressing (1) joint pain, (2) psoriasis arthritis and (3) the patient’s general well-being of psoriasis. Finally, two self-made questions were included about (1) side effects of treatment, and (2) screening for cardiovascular risk factors within the last year, including blood pressure (BP), blood glucose (BG), and blood lipids (BL). The content of each questionnaire and question can be seen at the website of the Danish Health Data Authority [11]. The national clinical coordination group additionally decided the algorithm to categorize and code PSO PROMs responses as a green, yellow, or red outcome. Green represents a response within a normal range, yellow represents a response that requires attention by the HCP, and red represents a response that requires immediate attention by the HCP. See Table S1 in the Additional file 1 for standard questionnaire scoring and colour-coding. The PSO PROMs where considered to have a status as red if just one of the questions or questionnaires in the PSO PROMs had answers that corresponded with the red code as defined by the national clinical coordination group.

### The PSO PROMs visitation study

For cases in which the patients had completed the PSO PROMs in advance of their in-person consultation, the study nurse asked the patient, the doctor, and the nurse involved in the consultation to identify the necessity of the consultation. This was done by asking verbatim; “*Do you believe …*” and then presenting the following statements as possible answers: 1) The consultation could have been postponed; 2) The doctor attending the consultation could have been replaced by a nurse; or 3) The scheduled consultation was appropriate. Additionally, the study nurse asked the patient, the doctor, and the nurse to comment on their selection, this was optional. The verbal answers to necessity of consultations were collected by the study nurse immediately after a consultation was finished. The patient, the doctor, and the nurse were asked separately to prevent anyone feeling obligated to give a specific answer.

The nurses are skilled within the field of psoriasis and with several years of experience. The nurses’ job is to assist the doctor within the consultation by measuring the patients’ blood pressure, and weight, support the patient in living with psoriasis, educate the patient in taking the medicine correctly and deliver the right amount of medicine the patients need to take home from the hospital. In this study the study nurse was the same as the nurse participating in the consultation and would also be the nurse who is suggested as possible replacement of the doctor in option number 2.

### Analysis

We used descriptive statistics to evaluate baseline characteristics of patients (see Table S2 in the Additional file 1), and the PSO PROMs responses coded as a green, yellow, or red outcome (see Table 1).

**Table 1:** PSO PROMs content and the patient results coded across red, yellow, and green outcomes

PSO PROMs content			
	Red	Yellow	Green
	n (%)	n (%)	n (%)
DLQI (Dermatology Life Quality Index)	26 (13,9)	50 (26,7)	111 (59,4)
PSSD sign (Psoriasis Sign Score)	7 (3,7)	33 (17,6)	147 (78,6)
PSSD symptom (Psoriasis Symptom Score)	5 (2,7)	29 (15,5)	153 (81,8)
BSA* (Body Surface Area)	-	-	-
MDI-2 (Major Depression Inventory)	32 (17,1)	20 (10,7)	135 (72,2)
PEST (Psoriasis Epidemiology Screening Tool)	21 (11,2)	16 (8,6)	150 (80,2)
Joint pain (Do you have joint pain?)	104 (55,6)	–	83 (44,4)
Psoriasis arthritis (Are you diagnosed with psoriasis arthritis?)	47 (25,1)	–	140 (74,9)
General well-being of psoriasis (How are your general well-being in regard to psoriasis?)	70 (37,4)	–	117 (62,6)
Cardiovascular screening (Have you had screening for cardiovascular risk factors within the last year (BG, BP, BL)?)	63 (33,7)	50 (26,7)	74 (39,6)
Side effects (Do you have side effects to the medicine?)	39 (20,9)	24 (12,8)	124 (66,3)

*BSA does not have a clear cut-off point. Mean score was calculated to 5,8 (range 0–61)

**Fig. 1:**
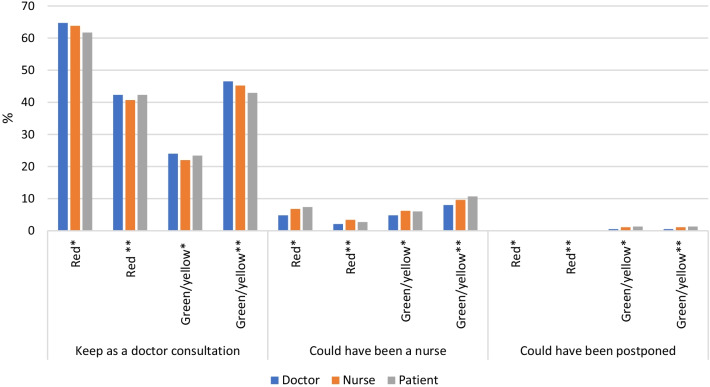
Doctor, nurse, and patient’s perceptions on required consultation and the patients’ PSO PROMs coded results. *All of PSO PROMs **Subset of PSO PROMs: DLQI, PEST, side effects & MDI-2

We also examined responses collected from the patient, doctor, and nurse identifying consultation needs and compared these to the PSO PROMs colour coded responses for each patient (see Fig. 1).

Two authors; AK and JJ used content analysis to examine patient, doctor, and nurse comments and created themes using inductive analysis. AK and JJ separately went through all the comments and each created immediate themes. The themes created where then compared and by consensus discussions the chosen themes were decided. This yielded a summary of reflections on the consultation needs and associated reasoning (see Table S3 in the Additional file 1).

Additionally, we did subgroup analyses to see if the participants with a PROM coded as red where different in age, gender, and medical treatment, from the participants with a PROM coded as green/yellow (see Table S4 in the Additional file 1).

## Results

The PSO PROMs were completed by 334 patients with psoriasis. A subset (n = 187, 56%) of patients participated in the PSO PROMs Visitation Study. The mean age of patients in the PSO PROMs Visitation Study was 52 years (range 18–83 years) and 96 (51.3%) patients in this sample identified as male. Within the Visitation Study group, 7% of patients used topical treatment only to manage their psoriasis symptoms. Other patients were treated using systemic (41.2%), biological (48.1%), or UVB light (1.6%) treatment. Four patients (2.1%) were treated with a combination of the methods listed above (see Table S2 in the Additional file 1).

### PSO PROMs responses

Our analysis of the PSO PROMs Visitation Study group demonstrated that 83% of the 187 patients showed at least one red outcome area in their PSO PROMs responses, demonstrating an immediate need for clinical intervention. The most common red outcome emerged from the patient identifying joint pain (55.6%), followed by low scores in the question about general well-being of psoriasis (37.4%), and whether screening for cardiovascular risk was performed within the last year (33.7%). The distribution of patients across assessments with red and yellow/green PSO PROMs outcomes is shown in Table 1.

### Need for consultation assessed by the doctor, nurse, and patient.

For the PSO PROMs Visitation Study, we collected 149 (79.7%) responses from patients, 187 (100%) responses from doctors, and 177 (94.7%) responses from nurses reporting the need for the consultation. The nurses’ responses were highly consistent (97.2% consistency) with the doctors’ responses. The patients’ responses were consistent with their doctor’s assessment in 93.3% of the cases. We found that the majority of in-person consultations with a doctor were deemed necessary by the patient (85.2%), doctor (88.8%), and nurse (85.9%). In 13% of nurse responses and 13.4% of patient responses, participants identified that consultations could have been carried out by a nurse, compared to only 9.6% of doctor responses. In seven cases the patient and doctor disagreed on the need for consultation. In six of these seven cases the doctors answered that the doctor consultation was necessary and in one case that it could have been a nurse consultation. In the seven cases only one patient deemed that the consultations should necessarily be with doctor, and five of the patients answered that the consultations could have been replaced by a nurse consultation and one consultation could have been postponed.

Most patients who met the inclusion criteria were invited to participate in the study and none declined. Patients was not asked if the study nurse was occupied due to other tasks (e.g. attending to another patient). Also, nurses did not provide a response, if they had not been present in the consultation. Comments to the choice of consultation need was optional.

Comparing the patient, doctor, and nurse responses addressing a need for consultation compared to the coded PSO PROMs results (red or green/yellow outcome), 23.4% of the patients with a green/yellow outcome were in need of a doctor’s consultation (see Fig. 1). Upon considering a subset of PSO PROMs questionnaires that reflect subjective responses (e.g., DLQI, PEST, MDI-2, and side effects), the proportion of patients that yielded a green/yellow outcome and were identified to require a doctor consultation increased to approximately 45% (see Fig. 1).

The responses to the question assessing the need for a consultation included 120 comments that were sorted into nine themes: 1. *New medicine/change in medicine*; 2. *Exacerbation of psoriasis symptoms;* 3. *Would like to talk to a doctor;* 4. *Physical examination/treatment of skin*; 5. *Control of new medicine/yearly follow-up*; 6. *Consultation due to joint pain;* 7. *Questions for the doctor about treatment*; 8. *Final consultation;* 9. *Miscellaneous.* Within these nine themes, most of these comments (32.1%) were categorised as *New medicine/change in medicine* (1), followed by (17.6%) *Control of new medicine/yearly follow-up with doctor* (5). Each theme is described in Table S3 in the Additional file 1. The majority of these comments referred to patients with PSO PROMs responses coded as red outcomes, and this accounted for 92 comments (76.7%). Patients with responses coded as green/yellow outcomes only accounted for 28 of these comments (23.3%) (see Table S3, Additional file 1). Comments to the choice of consultation was optional and only given by 120 patients.

### Subgroup analysis of patients.

In the subgroup analysis we found a tendency towards the patients with a PROM or subset of PROM coded as red were younger, more often female, and more often treated with systemic and biological than the group of patients with a PROM or a subset of PROM coded as yellow/green. The subgroup analysis can be seen in Table S4 in the Additional file 1.

## Discussion

Overall, this study shows that this preliminary set of PSO PROMs alone is not a reliable source for identifying patient consultation needs. We found that 23.4% of patients with green/yellow outcomes still required a doctor consultation (according to the patient, doctor, and nurse) when all sections of the PSO PROMs were evaluated. During this study, a government change in regulations impacted psoriasis treatment, where all patients undergoing adalimumab treatment were transitioned to biosimilar biologic treatment. To account for this disruption in our study design, we evaluated questionnaires that reflect subjective accounts of patient well-being (e.g., DLQI, PEST, side effects, and MDI-2) separately from the full PSO PROMs. The proportion of patients with green/yellow outcomes that required a doctor consultation increased to 42.9% of patients using only the subset PSO PROMs sections. For this subset of PSO PROMs there is also a slight increase (from 6.0% to 10.7%) in patients’ who thought they could have been seen by a nurse instead of a doctor. However, the increase in number of patients with a green/yellow outcome who still feels it is necessary to see a doctor indicates that we, with the PSO PROM are not able to measure the patient’s individual needs in regard to consultations. It is difficult to comprehensively assess the impact of living with a skin condition and Pattinson [16] describes that no single set of PROMs fully captured the patient experience.

In the subgroup analysis we found that the patients with a PROM or subset of PROM coded as red, was only slightly younger but more often female and more often treated with systemic and biological medicine as opposed to the patients with a PROM or subset of PROM coded as yellow/green. We do not know the reason for this distribution, but in a study of German and Swiss psoriasis registries, it is shown that women are rating some symptoms (e.g. feeling of depression, sleep quality and everyday productivity) to be significantly more important than men [17]. This could be a possible explanation for the higher proportion of women with PROM or subset of PROM coded as red. The treatment distribution might reflect the fact that the patients with a PROM coded as red have a more severe degree of psoriasis.

Although the patient, doctor, and nurse consultation responses were consistently in favour of a doctor consultation, it is important to consider that this approach may be considered the “normal practice” and therefore may reflect a default response by patients, doctors, and nurses. In a related survey carried out within the primary care setting, patients often report a preference for seeing a doctor over a nurse [18]. Patients may perceive a difference in skill and knowledge between nurses and doctors, and therefore feel more confident in their care plan following a doctor consultation. This notion is reflected in this study’s recorded comments, where 13% of patients stated that they needed to see a doctor or had questions specifically for the doctor. There is also a possibility of the patient, doctor, and nurse not knowing what to expect from a nurse consultation. Future work may explore whether alternative clinic standards would challenge these assumptions. For example, within the area of diabetes management, nurse consultations have been well integrated to the provision of care for many years. Both doctors and nurses have individual patient consultations with the use of PROMs and the results of recent both qualitative and quantitive studies show patients well satisfied with their care [19, 20]. A follow-up study within the field of psoriasis, may examine whether patients who engage in consultations with nurses when their disease is under control report a similar quality of care to patients who only attend doctor consultations.

Studies that explore the broader uses of PROMs in patient care suggest that PROMs may serve as a tool to guide clinicians towards a range of treatment decisions, for example additional pharmacological support, lifestyle recommendations, and referral to other experts [21, 22]. This idea was considered in a recent oncology study that created guidelines for the clinician to navigate in response to issues identified by PROMs. The purpose of these guidelines is to help clinicians best use information captured by PROMs and point them towards a range of resources they might recommend to their patients. This offers a structured method for clinicians to seek out broader support for their patients, including access to other HCPs as needed [22]. For example, in England PROMs have been used to decide when patients with hip osteoarthritis qualify for surgery by tracking changes to PROMs over time. Although PROMs are not the sole determinants of a patient’s treatment plan, the addition of predictive validity and longitudinal tracking can provide valuable data to guide clinician decision-making [10]. It is possible to imagine that a longitudinal tracking of a patients PSO PROMs would be more beneficial as it could show the progress and regression for the patient instead of a snapshot solely evaluated on the base of standard clinical algorithms. Despite efforts to integrate these measures into healthcare settings, PROMs need to be further developed to ensure they are recording relevant and useful patient data that inform clinical decisions.

The PSO PROMs used in this study were evaluated by both patients and HCPs, who reported they were generally satisfied with using this tool in patient care. Nevertheless, more specific, and well-tested guidelines are needed to support clinicians and patients and to identify whether PROMs can inform consultation needs. Additionally, further investigation will refine the selection of questions that is disseminated within the PSO PROMs to identify patient visitation needs most accurately. A recent qualitative study exploring PROMs’ impact on patient-clinician interactions describes how PROMs can be used to guide dialogue addressing psychosocial problems. PROMs facilitate patient-centred communication addressing emotions, fears, and concerns that are not captured in regular consultations [21]. This work suggests that PROMs also function as a resource to address a wider range of patient concerns. The scope of clinician consultations is broadened by integrating PROMs into healthcare practices [21] and HCPs need to prioritize a range of patient concerns in order to maintain patient satisfaction and a patient-centred approach to care.

PROMs have been increasingly researched and implemented in healthcare during the last decade and we expect the research and knowledge about the best use of PROMs for both patients and HCP will continue to rise. Our study contributes to this research, by making us critically reflect upon how PSO PROM can be used in its current form for visitational support with patients diagnosed with psoriasis.

## Limitations

We noted a handful of limitations in this study that are necessary to consider when interpreting our findings. Firstly, not all patients (20.3%) included in the Visitation Study identified which consultation (doctor, nurse, postponed) was currently required. This is a considerable proportion of missing responses and it is impossible to know these patients’ current needs. Secondly, some patients may have been reluctant to openly share their perspective on whether the doctor consultation could have been replaced by a nurse consultation. Although this is a possibility, most patients reported that a doctor’s consultation was needed.

Finally, all comments in the patient journal were recorded by the study nurse, however they did not always indicate whether the comment was made by the patient, the doctor, or the nurse. It is currently unknown which individual (patient, doctor, or nurse) made each comment, which formed the basis of the themes.

## Conclusion

Psoriasis PROMs were supportive in the consultation as it helped to enhance patient and HCP communication to what was experienced as meaningful for the patient. However, this study was the first to explore the use of PSO PROMs as visitational support and to identify which type of consultation is required for psoriasis patients. The findings suggest that the current PSO PROMs cannot sufficiently guide HCPs to determine what kind of consultation is needed. The patient, doctor, and nurse perspectives on consultation needs do not align with the patient’s PSO PROMs responses. Ongoing research needs to further investigate the use of PSO PROMs to inform patient consultation needs.

## Supplementary Information

**Additional file 1. Table 1**. The contents and coding algorithms applied to PSO PROMs.** Table 2**. Baseline characteristics of the 187 PSO PROMs respondents.** Table 3**. The 120 comments from patients, doctors, and nurses sorted across nine themes.** Table 4**. The subgroup analyses of patients with a PROM coded as red or yellow/green.

## Data Availability

The datasets used and/or analysed during the current study are available from the corresponding author on reasonable request.

## Abbreviations

BSA: Body Surface Area
BG: Blood glucose
BL: Blood lipid
BP: Blood pressure
DLQI: Dermatology Life Quality Index
HCP: Healthcare professionals
MDI-2: Major Depression Inventory
PEST: Psoriasis Epidemiology Screening Tool
PROM: Patient reported outcomes
PSO: Psoriasis
PSSD: Psoriasis Symptoms and Signs Score

## References

[1] Danielsen K (2019). Prevalence of psoriasis and psoriatic arthritis and patient perceptions of severity in Sweden, Norway and Denmark: results from the Nordic patient survey of psoriasis and psoriatic arthritis. Acta Derm Venereol.

[2] Khoury LR (2018). A prospective 52-week randomized controlled trial of patient-initiated care consultations for patients with psoriasis. Br J Dermatol.

[3] Griffiths CEM (2021). Psoriasis. Lancet.

[4] Wan MT (2020). Anticipated and perceived stigma among patients with psoriasis. J Psoriasis Psoriatic Arthritis.

[5] Wu JJ (2017). Contemporary management of moderate to severe plaque psoriasis. Am J Manag Care.

[6] Khoury LR, Skov L, Møller T (2017). Facing the dilemma of patient-centred psoriasis care: a qualitative study identifying patient needs in dermatological outpatient clinics. Br J Dermatol.

[7] Doolin JW (2020). Why focus on patient-reported outcome measures in older colorectal cancer patients?. Eur J Surg Oncol.

[8] Brix ATH (2021). Patient-reported outcome measures for angioedema: a literature review. Acta Derm Venereol.

[9] Basch E (2018). Implementation of patient-reported outcomes in routine medical care. Am Soc Clin Oncol Educ Book.

[10] Black N (2013). Patient reported outcome measures could help transform healthcare. BMJ.

[11] sekretariatet, P. *Psoriasis*. [webpage] 2021 [cited 2022 20/01–2022]; Available from: https://pro-danmark.dk/da/omraader/psoriasis.

[12] Finlay AY, Khan GK (1994). Dermatology Life Quality Index (DLQI)–a simple practical measure for routine clinical use. Clin Exp Dermatol.

[13] Feldman SR, Mathias SD, Schenkel B, Colwell HH, McQuarrie K, Randazzo B, Han C (2016). Development of a patient-reported outcome questionnaire for use in adults with moderate-to-severe plaque psoriasis: the psoriasis symptoms and signs diary. J Dermatol Dermatol Surg.

[14] Olsen LR (2003). The internal and external validity of the Major Depression Inventory in measuring severity of depressive states. Psychol Med.

[15] Ibrahim GH (2009). Evaluation of an existing screening tool for psoriatic arthritis in people with psoriasis and the development of a new instrument: the Psoriasis Epidemiology Screening Tool (PEST) questionnaire. Clin Exp Rheumatol.

[16] Pattinson RL (2021). Patient-reported outcome measures in dermatology: a systematic review. Acta Derm Venereol.

[17] Maul JT (2019). Gender and age significantly determine patient needs and treatment goals in psoriasis—a lesson for practice. J Eur Acad Dermatol Venereol.

[18] Paddison CAM (2018). What happens to patient experience when you want to see a doctor and you get to speak to a nurse? Observational study using data from the English General Practice Patient Survey. BMJ Open.

[19] Haugstvedt A (2021). Nurses' and physicians' experiences with diabetes consultations and the use of dialogue tools in the DiaPROM pilot trial: A qualitative study. Diabet Med.

[20] Laurberg T (2022). Randomized controlled study to evaluate the impact of flexible patient-controlled visits in people with type 1 diabetes: The DiabetesFlex Trial. Diabet Med.

[21] Mejdahl CT (2020). Patient-reported outcome measures in the interaction between patient and clinician—a multi-perspective qualitative study. J Patient Rep Outcomes.

[22] Hughes EF (2012). What can I do? Recommendations for responding to issues identified by patient-reported outcomes assessments used in clinical practice. J Support Oncol.

