# Supplementary material for: Can patient-reported outcome measures be used to predict consultation needs in patients with psoriasis?: A survey study
Source: J Patient Rep Outcomes. 2022 Jul 23;6:81. doi: 10.1186/s41687-022-00490-7 (PMC9308841; doi:10.1186/s41687-022-00490-7)
Supplement: Supplementary file 1 — Additional file 1. Table 1. The contents and coding algorithms applied to PSO PROMs. Table 2. Baseline characteristics of the 187 PSO PROMs respondents. Table 3. The 120 comments from patients, doctors, and nurses sorted across nine themes. Table 4. The subgroup analyses of patients with a PROM coded as red or yellow/green. [file 41687_2022_490_MOESM1_ESM.docx]

**Supplementary Material**

**Table 1**. The contents and coding algorithms applied to PSO PROMs

| **Questionnaires & questions** | **The coding algorithms applied to the questionnaires and questions*** | | |
| --- | --- | --- | --- |
|  |  |  |  |
|  | **Green** | **Yellow** | **Red** |
| **DLQI** | <5 | ≥ 5 | ≥10 |
| **PSSD symptom** | <40 | 40-80 | >80 |
| **PSSD sign** | <40 | 40-80 | >80 |
| **BSA**** | - | - | - |
| **Joint pain** | No |  | yes |
| **Psoriasis arthritis** | The answer “no” opens up to PEST |  | Yes |
| **PEST** | <3 | 3 | >3 |
| **Cardiovascular screening** | Yes | Don’t know | no |
| **Side-effects** | No | Don’t know | yes |
| **MDI-2** |  | <4 | ≥4 |
| **General well-being** | <4 |  | ≥4 |

*Green = within normal range, Yellow = requires attention by the HCP, Red= requires immediate attention by the HCP.

** BSA does not have a clear cut-off point, therefore the number of palms was always coded as green.

**Table 2.** Baseline characteristics of the 187 PSO PROMs respondents

| **Characteristic** | **Answers of PSO PROMs** |
| --- | --- |
| **Sex, n (%)** |  |
| Male | 96 (51,3) |
| **Age, Mean (range)** | 52 (18-83) |
| **Registration of treatment, n (%)** |  |
| Topical (only) | 13 (7) |
| Systemic | 77 (41.2) |
| Biological | 90 (48.1) |
| UVB | 3 (1.6) |
| Topical and UVB | 1 (0.5) |
| Systemic and UVB | 2 (1.1) |
| Systemic and Biological | 1 (0.5) |

**Table 3.** The 120 comments from patients, doctors, and nurses sorted across nine themes.

|  |  | **Full PSO PROMs** | | **Subset of PSO PROMs: DLQI, PEST, side effects & MDI-2** | |
| --- | --- | --- | --- | --- | --- |
| **Themes** | **Total**  **n (%)** | **Red**  **n (%)** | **Green/yellow**  **n (%)** | **Red**  **n (%)** | **Green/yellow**  **n (%)** |
| **1. New medicine/change in medicine** | 42 (32.1) | 30 (22.9) | 12 (9.2) | 21 (16.0) | 21 (16.0) |
|  |  |  |  |  |  |
| **2. Exacerbation of psoriasis** | 12 (9.2) | 11 (8.4) | 1 (0.8) | 6 (4.6) | 6 (4.6) |
|  |  |  |  |  |  |
| **3. Would like to talk to a doctor** | 12 (9.2) | 6 (4.6) | 6 (4.6) | 6 (4.6) | 7 (5.3) |
|  |  |  |  |  |  |
| **4. Physical examination / treatment of skin** | 12 (9.2) | 12 (9.2) | 0 (0) | 6 (4.6) | 6 (4.6) |
|  |  |  |  |  |  |
| **5. Control of new medicine / yearly follow up** | 22 (17.6) | 15 (11.5) | 7 (5.3) | 6 (4.6) | 15 (11.5) |
|  |  |  |  |  |  |
| **6. Consultation due to joint pain** | 6 (4.6) | 6 (4.6) | 0 (0) | 4 (3.1) | 2 (1.5) |
|  |  |  |  |  |  |
| **7. Questions for the doctor about disease or treatment** | 5 (3.8) | 5 (3.8) | 0 (0) | 3 (2.3) | 2 (1.5) |
|  |  |  |  |  |  |
| **8. Final consultation** | 4 (3.1) | 3 (2.3) | 1 (0.8) | 3 (2.3) | 1 (0.8) |
|  |  |  |  |  |  |
| **9. Miscellaneous** | 5 (3.8) | 4 (3.1) | 1 (0.8) | 3 (2.3) | 2 (1.5) |
|  |  |  |  |  |  |
| **Total** | **120 (100)** | **92 (76.7)** | **28 (23.3)** | **60 (50)** | **62 (51.7)** |

*Description of each theme:

1. Related to comments about/from patients who had either systemic or biological medicine described or needed to have their dose of the medicine adjusted due to lack of effect, side effects etc.
2. Comments about/from patients who experienced a flare up in their psoriasis.
3. Describes a group of patients who said that they felt more comfortable seeing the doctor or said that they just liked seeing the doctor.
4. Consists of comments about an element the patient had to have evaluated or if the patient were treated in clinic with for example injections, bandages, occlusion, or other treatments.
5. Comments relating to consultation being a check-up after start of new medicine or the once yearly control where blood pressure, weight and specific blood test are checked.
6. Described patients who had pain in their joints and where an evaluation of psoriasis arthritis was done.
7. This theme consisted of comments specifying that the patient had questions specifically for the doctor.
8. Final consultation was used for comments from/about patients about being discharged from the outpatient clinic.
9. Comments related to one patient having psychiatric diagnosis and needing stability, and four related to compliance issues.

**Table 4.** The subgroup analyses of patients with a PROM coded as red or yellow/green.

|  | Patients with a PROM coded  as red | Patients with a PROM coded as yellow/green | Patients with a subset of PROM coded as red | Patients with a subset of PROM coded as yellow/green |
| --- | --- | --- | --- | --- |
| **Patients, n** | 132 | 55 | 82 | 105 |
| **Sex, n (%)** |  |  |  |  |
| Male | 61 (46.2) | 35 (63.6) | 40 (48.8) | 56 (53.3) |
| **Age, Mean (range)** | 51.6 (18-81) | 53,9 (20-78) | 49.6 (18-81) | 54.3 (20-78) |
| **Registration of treatment, n (%)** |  |  |  |  |
| Topical (only) | 11 (8.3) | 2 (3.6) | 6 (7.3) | 7 (6.7) |
| Systemic | 51 (38.6) | 26 (47.3) | 33 (40.2) | 44 (41.9) |
| Biological | 63 (47.7) | 27 (49.1) | 33 (40.2) | 44 (41,9) |
| UVB | 3 (2.3) | 0 (0) | 1 (1.2) | 2 (1.9) |
| Topical + UVB | 1 (0.75) | 0 (0) | 1 (1.2) | 0 (0) |
| Systemic + UVB | 2 (1.5) | 0 (0) | 2 (2.4) | 0 (0) |
| Systemic + biological | 1 (0.75) | 0 (0) | 0 (0) | 0 (0) |
